# Investigating the factors influencing students’ academic enthusiasm for a shift of paradigm among education managers shaping academic pedagogy

**DOI:** 10.1186/s12909-023-04453-4

**Published:** 2023-06-27

**Authors:** Javad Moghadasi, Leila Keikavoosi-Arani

**Affiliations:** 1 Department of Higher Education Administration, School of Management and Economics, Science and research branch, Islamic Azad University, Tehran, Iran; 2 Department of Healthcare Services Management, School of Health, Research Center for Health, Safety and Environment, Alborz University of medical sciences, Karaj, Iran

**Keywords:** Academic enthusiasm, Improvement, Paradigm, Pedagogy

## Abstract

**Background:**

Education managers are among the most important determinants of a university’s academic pedagogy and plans to improve the quality of education. To improve the quality of education and academic enthusiasm of students in a medical university, it might be necessary to improve the university’s pedagogy paradigm through people who shape this pedagogy. This study aimed to investigate the factors that influence the academic enthusiasm of students in a medical university with the purpose of improving the university’s pedagogy paradigm.

**Methods:**

This cross-sectional descriptive-correlational study was conducted on the students of Alborz University of Medical Sciences in the academic year 2021–2022 (3180 students). The sample size was calculated to be 343 using Cochran’s formula. The participants were recruited by stratified random sampling with respect to the proportion of students in different faculties, disciplines, and education levels. The data collection tool was a researcher-made questionnaire. Data were analyzed by one-sample t-test and confirmatory factor analysis using SMART-PLS and SPSS26.

**Results:**

The developed model consisted of 3 dimensions (academic, individual and social), 10 components (teaching-learning environment, culture, extracurricular activities, facilities and equipment, attitude, knowledge, skill, classmate, family, relatives and friends) and 64 indicators.

**Conclusion:**

The developed model can help medical universities take a step towards improving the academic productivity and performance of their students and gain a competitive advantage in this respect.

## Background

Academic pedagogy is a subject of keen interest to institutions of higher education [1, 2]. Indeed, universities have to plan to maintain and improve the quality of education they provide to students [3]. One of the indicators of this quality is academic enthusiasm [4]. Bakker et al. (2008) define academic enthusiasm as a set of behaviors concerning learning and academic development which represent the quality of effort that learners put into targeted educational activities to directly contribute to achieving the desired outcomes [5]. Pomerantz describes academic enthusiasm as a new paradigm that constitutes the basis of values and beliefs that guide learners to reach their goals. According to Pomerantz, this paradigm is a new step toward the evolution of student activities and promotes a new way of thinking about how much students engage in activities and behaviors outside the classroom that can affect their learning [6]. Academic enthusiasm can also be described as the amount of energy that learners spend to carry out academic activities effectively and efficiently [7]. In contemporary pedagogy, students are seen as important active participants in the learning process and it is believed that educational goals can only be achieved when students properly play their role [8]. In general, more enthusiastic students tend to have better academic performance [9] and academic motivation [10] on account of being more hopeful and having a higher sense of self-regulation. These students have a greater presence in the classroom, which is the most important setting for scientific education [11, 12], interact more with professors and other students, more actively participate in learning by asking questions and giving suggestions, and are generally more successful in education [13]; Success is one of the needs of every human being [14]. Enthusiastic students study more, have more academic satisfaction, are more likely to graduate, exhibit greater flexibility in solving problems, are more adaptable, and have a stronger drive for learning [15, 16]. According to Thomas Kuhn’s definition of “paradigm”, this concept refers to a specific prevailing way of thinking and problem-solving among the members of a scientific field, which represents their shared commitments, beliefs, values, methods, and viewpoints [17]. Without a change of paradigm among education managers who shape a university’s pedagogy, it might be impossible to improve students’ academic enthusiasm. Also, considering the extremely interesting educational implications of academic enthusiasm, access to an academic enthusiasm model will allow education managers, who are among the most important determinants of academic pedagogy, to plan better for improving the quality of education. However, for such a model to be effective, it needs to match the rules, needs, infrastructure, resources, facilities, planning system, and conditions of the university where it is going to be implemented. In this study, the authors tried to identify the factors that influence the academic enthusiasm of the students of Alborz University of Medical Sciences in order to improve the university’s pedagogy paradigm. Alborz University of Medical Sciences is a public university based in Iran’s Alborz province, which operates under the supervision of the country’s Ministry of Health and Medical Education [18]. This university offers a wide range of education programs, with admittance awarded through Iran’s nationwide university entrance exam. The university is one of the top choices for talented students who earn high ranks in the nationwide university entrance exam. The university’s location allows students to interact with the country’s other major universities and use the facilities located in the capital city (Tehran). Considering the relatively good academic ranking of this university among Iranian institutions of medical education, improving the university’s pedagogy paradigm could be a major step toward enhancing the academic performance of future Iranian healthcare professionals.

## Methods

### Study design and participants

This cross-sectional descriptive-correlational study was conducted on the population of students receiving education in any discipline at any level in Alborz University of Medical Sciences’ faculties. There were 3180students at the time of this study. School of medicine with 1267 students, School of dentistry with 358 students, School of pharmacy with 278 students, School of nursing with 455 students, School of allied medical sciences with 492 students, School of health with 330 students in the academic year 2021–2022 (N = 3180).

### Sample size

The sample size was calculated by Cochran’s formula, according to which the minimum appropriate sample size was determined to be 343 people. Considering the possibility of dropouts, a total of 360 students were recruited.

### Sampling methods

The participants were recruited by stratified random sampling with each faculty treated as one stratum and with respect to the proportion of students in different disciplines and education levels. In order to comply with the appropriate ratio of students in different fields of study and different levels of study, the required number of samples was medicines (143 students), dentistry (41), pharmacies (31 students), nursing (51 students), allied medical sciences (56 students), health (38students).Sampling in each field of study was simple random.

### Ethics

A researcher obtained the surnames and contact numbers of the representatives of the students in different fields and levels of all entries from the head of the education unit of each faculty and contacted each of them and was informed of the time and day of their classes. After getting permission from the professor, she appeared in the class and fully explained the objectives and methods of the research, and then she gave the questionnaire only to the students who wanted to complete the questionnaire and gave informed consent. He gave 20 minutes to complete and then collected the questionnaire. This work continued until the completion of the number of samples in different fields of study and at different levels of study. Then he gave the questionnaire only to those students who were willing and satisfied to complete the questionnaire. After initial data screening to remove inadequate samples (e.g. people who did not completely fill out the questionnaires), the total number of samples was reduced to 355 people.

### Inclusion and exclusion criteria

The inclusion criteria were providing informed consent and having finished at least one semester in the university’s bachelor’s, master’s, or doctoral programs. The exclusion criteria were not answering the questionnaires and being a new, dropout, expelled, or graduated student.

### Data collection tool

**Fig. 1:**
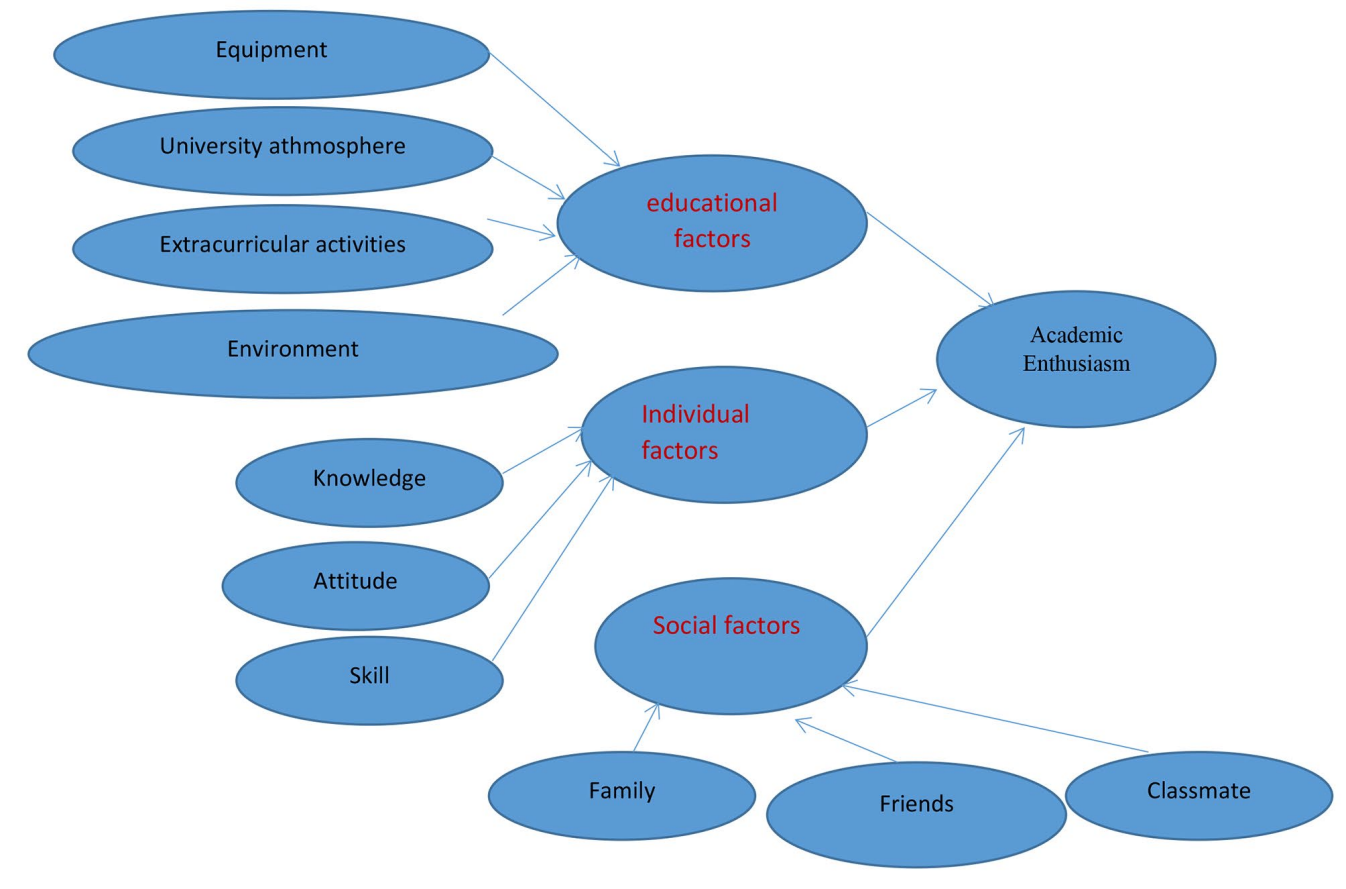
Conceptual Model

The data collection tool was a researcher-made questionnaire, which was created after reviewing the literature and similar studies in Persian and English such as dissertations, books, translations, articles in accordance with the research goals and questions by searching at PubMed, Web of Science, SCOPUS, Pro Quest, Science Direct, Emerald and Google Scholar databases [19–27,…]. The keywords included academic engagement, educational settings, student, and enthusiasm. In addition, a general search was performed in the Google search engine for “academic engagement instrument”. Finally, several items of two measures were utilized in the initial item pool and based on that, the theoretical framework of the research (conceptual model) was designed and in this model (Fig. 1) academic enthusiasm was considered as a dependent and hidden variable. Other factors are considered as independent and hidden variables of the theoretical model. This questionnaire consisted of two parts. In the first part, after a brief explanation about the questionnaire’s general purpose, the importance of answering truthfully to the questions, and the confidentiality of the collected data, the respondents were asked to provide their personal and demographic information, including age, gender, marital status, field of study, and education level. The second part of the questionnaire consisted of 78 close-ended questions, each with five responses on the five-point Likert scale: Very Low, Low, Moderate, High, and Very High. The responses were scored from 1 for “Very Low” to 5 for “Very High”. Of these 78 questions, questions 1–42 aimed to measure educational factors, questions 43–59 intended to measure individual factors, and questions 60–78 aimed to measure social factors. Both qualitative and quantitative methods were used to determine content validity. In the qualitative method, the prepared questionnaire was given to 10 professors and experts in the fields of healthcare services management (1 people), education management (3 people), higher education management (4 people), and medical education (2 people). At this stage, they were asked to check the questionnaire based on the use of appropriate words, the placement of items in the appropriate place, and compliance with grammar. To check content validity quantitatively, content validity ratio (CVR) and content validity index (CVI) were used. In order to determine the content validity ratio, experts were asked about the necessity of each item, and values higher than 0.62 were accepted based on the Lavshe table. To determine the content validity index, the criteria of relevance, clarity and simplicity of each item were examined and values higher than 0.79 were accepted. After determining content validity, 10 questions were removed from the questionnaire. In the next step, in order to determine the clarity of the items, the questionnaire was given to 15 students to read it and answer it, and ask any questions or ambiguities in understanding the items. Based on the comments and suggestions received from the mentioned people, the necessary changes were made to clarify the items. Also, in this step, in order to remove unimportant and inappropriate items, the quantitative method of impact score was used. An impact score above 1.5 was considered acceptable. According to the students’ comments, 4 questions were removed from the questionnaire. Face validity was established by asking the experts to comment on the appearance, comprehensibility, adequacy, ambiguity, form, order, and number of questions and then addressing the raised issues by adding, removing, or changing the questions accordingly.

### Final questionnaire

Finally, the number of questions in the questionnaire reached 68 questions. Questions 1–35 aimed to measure academic factors (with the Cronbach’s alpha coefficient: 0.962), questions 36–51 intended to measure individual factors (with the Cronbach’s alpha coefficient: 0.943) and questions 52–68 aimed to measure social factors (with the Cronbach’s alpha coefficient: 0.93). The questionnaire of academic enthusiasm was measured by explicit variables as follows:

- Facilities and equipment was assessed by 6 questions e.g., “The educational facilities of the university including video projectors, teaching aids and appropriate chairs are effective in engaging me in my studies”, “The existence of all kinds of well-equipped laboratories in this university are effective in engaging me in my studies”. The Cronbach’s alpha coefficient calculated for this section was 0.892.
- Culture was assessed by 11 questions e.g., “Considering the opinions of students in the decision-making of managers and professors is effective in engaging me in my studies”, “The behavior and non-discrimination of professors, administrators and university staff with students is effective in my engaging me in my studies”. The Cronbach’s alpha coefficient for this part was 0.894.
- Extracurricular activities was assessed by 7 questions e.g., “The existence of active scientific and cultural associations in the university is effective in my engaging me in my studies”, “Organizing appropriate scientific conferences in the university is effective in my engaging me in my studies”. The Cronbach’s alpha coefficient for this part was 0.892.
- Teaching-learning environment was assessed by 11 questions e.g., “Fair and accurate evaluation of students by professors is effective in my engaging me in my studies”, “Giving equal opportunity and having a free-thinking space for expressing opinions in the classroom in the university is effective engaging me in my studies”. The Cronbach’s alpha coefficient for this part was 0.935.
- Knowledge was assessed by 4 questions e.g., “Having the necessary knowledge about my future career after obtaining a university degree in the field of study is effective in my engaging me in my studies”, “The alignment of knowledge and information of myself and my professors with my field of study is effective in engaging me in my studies”. The Cronbach’s alpha coefficient for this part was 0.870.
- Attitude was assessed by 8 questions e.g., “The sense of responsibility and my role in the economic cycle of the family is effective in engaging me in my studies”, “My beliefs and my beliefs about my field of study is effective in engaging me in my studies”. The Cronbach’s alpha coefficient for this part was 0.9.
- Skill was assessed by 4 questions e.g., “Having the ability to plan carefully to study course materials during the semester is effective in engaging me in my studies”, “Having problem solving skills is effective in engaging me in my studies”. The Cronbach’s alpha coefficient for this part was 0.834.
- Family was assessed by 6 questions e.g., “The necessity and value of continuing education in terms of family culture is effective in engaging me in my studies”, “Supporting and encouraging the family to study and strive in education is effective in engaging me in my studies”. The Cronbach’s alpha coefficient for this part was 0.910.
- Relatives and friends was assessed by 4 questions e.g., “Having educated relatives and family friends is effective in engaging me in my studies”, “Living conditions and well-being of educated relatives and acquaintances is effective in engaging me in my studies”. The Cronbach’s alpha coefficient for this part was 0.858.
- Classmate was assessed by 7 questions e.g., “Helping my classmates to solve my problems is effective in engaging me in my studies”, “Scientific books and articles of my classmates regarding subjects related to my field of study is effective in engaging me in my studies”. The Cronbach’s alpha coefficient for this part was 0.854. The Cronbach’s alpha coefficient for this part was 0.93 (Table 4).

All the questions in the questionnaire were stated positively. Construct validity was established by conducting a factor analysis (Four items from the variable “Culture” deleted). Five responses on the five-point Likert scale: Very Low, Low, Moderate, High, and Very High. The responses were scored from 1 for “Very Low” to 5 for “Very High”. The reliability of the questionnaire was determined by a pilot administration among 30 students, from whom 28 completed forms were collected. The reliability of the questionnaire was done with Cronbach’s alpha and test-retest on a group of 30 students. The amount of Cronbach’s alpha equal to or more than 0.7 was considered suitable, which ranged from 0.85 to 0.96 in the constructs of the questionnaire. The test-retest reliability of questionnaire constructs was obtained from 0.83 to 0.9.

### Statistical analysis

After establishing the validity and reliability of the research tool, the finalized questionnaire was distributed among eligible students. After establishing the validity and reliability of the research tool, the finalized questionnaire was distributed among eligible students. The collected data were analyzed by descriptive statistics (frequency, percentage, mean, standard deviation, and variance), and inferential statistics (one-sample t-test, confirmatory factor analysis) using SPSS v.26 and SMART-PLS v.3.3.

## Results

As the results of Table (1) show, 211 (59.4%) out of the 355 participating students were women and 144 (40.6%) were men. Also, out of the 355 participants, 5 (1.4%) were Associate of Science (ASc) students, 27 (7.6%) were discontinuous Bachelor of Science (BSc) students, 112 (31.6%) were continuous BSc students, 13 (3.7%) were Master of Science (MSc) students, 189 (53.2%) were Doctor of Medicine (MD) students, and 9 (2.5%) were MDs participating in specialty training programs. As can be seen, the majority of the respondents were MD students.

**Table 1:** Demographic characteristics of participants (N = 355)

Variables	Group	Percent	Frequency
**Sex**	Female	59.4	211
	Male	40.6	144
	Total	100.0	355
**Age(y)**	18–25	67.0	238
	26–30	11.8	42
	31–35	10.1	36
	36–40	7.9	28
	41–50	2.0	7
	> 50	1.1	4
	Total	100.0	355
**Marital status**	Married	19.2	68
	Single	80.8	287
	Total	100.0	355
**Education**	Ph.D.	2.5	9
	Doctor of Medicine(MD)	53.2	189
	Master of Science (MSc)	3.7	13
	Bachelor of Science(Continuous)	31.6	112
	Bachelor of Science(Discontinuous)	7.6	27
	Associate of science degree	1.4	5
	Total	100.0	355
**Job**	employed	34.1	121
	Unemployed	65.9	234
	Total	100.0	355
**Parent Education**	Ph.D.	10.1	36
	Diploma&Associate Degree	42.5	151
	Masters	20.0	71
	Bachelor’s degree	27.3	97
Total		100.0	355

A total of 355 valid and analyzable questionnaires were collected. Outer models were assessed in terms of three criteria: reliability, convergent validity, and divergent validity. For reliability assessment, reliability had to be checked at the indicator level and the latent variable level. Indicator reliability was assessed by measuring factor loadings, and latent variable reliability was checked through composite reliability. Indicator reliability is the square of the items’ factor loadings and needs to be at least 0.5, meaning that at least half of the variance of the indicator is explained by the latent variable. Therefore, factor loadings of 0.7 or higher are desirable, loadings below 0.4 are unacceptable, and loadings between 0.4 and 0.7 can be included in the model if convergent validity (AVE) is not lower than 0.5. The results in relation to factor loadings are presented in Table 2. Four items from the variable “Culture” that had a factor loading below 0.6 were excluded from the model. All other factor loadings were higher than 0.6. As mentioned, latent variable reliability was checked through composite reliability. The results regarding the model’s divergent validity are presented in Table 2.

**Table 2:** Measurement Model, Collinearity Statistics and descriptive statistics

Constructs		Item	Measurement Model			Collinearity	descriptive statistics			
			Loading Factor	S.E	T	VIF	Mean	Std.D	Skewness	Kurtosis
First Order Constructs	Facilities and equipment	X01	0.756	0.032	23.437	2.347	3.559	1.153	-0.597	-0.346
		X02	0.846	0.019	45.272	2.948	3.977	1.134	-1.116	0.581
		X03	0.842	0.022	38.098	3.24	3.763	1.107	-0.889	0.346
		X04	0.856	0.02	42.672	3.491	3.901	1.109	-1.028	0.533
		X05	0.819	0.023	35.927	2.212	4.161	1.026	-1.434	1.840
		X06	0.717	0.034	21.071	1.744	3.571	1.254	-0.560	-0.602
	Culture	X07	0.686	0.046	14.933	1.544	4.175	1.036	-1.354	1.487
		X11	0.717	0.045	15.935	2.337	4.094	1.040	-1.227	1.050
		X12	0.75	0.044	16.856	2.496	4.178	1.047	-1.334	1.241
		X14	0.829	0.031	27.055	2.354	4.088	0.995	-1.222	1.304
		X15	0.807	0.025	32.057	2.277	3.971	1.116	-0.978	0.238
		X16	0.818	0.041	19.807	2.69	4.117	1.042	-1.256	1.105
		X17	0.858	0.026	33.18	3.104	4.108	1.003	-1.245	1.369
	Extracurricular activities	X18	0.705	0.044	15.914	1.582	4.082	1.011	-1.215	1.235
		X19	0.674	0.044	15.318	1.803	3.264	1.353	-0.260	-1.097
		X20	0.765	0.033	22.955	2.085	3.497	1.247	-0.467	-0.783
		X21	0.846	0.026	32.313	3.306	3.733	1.231	-0.830	-0.205
		X22	0.807	0.031	25.645	2.802	3.797	1.254	-0.894	-0.178
		X23	0.845	0.021	41.08	2.791	3.624	1.217	-0.646	-0.447
		X24	0.801	0.031	25.549	2.41	3.582	1.164	-0.499	-0.453
	Teaching-learning environment	X25	0.783	0.04	19.415	2.849	4.099	0.938	-1.091	1.107
		X26	0.866	0.019	46.582	4.252	4.231	0.968	-1.498	2.236
		X27	0.816	0.029	28.495	2.795	4.256	1.003	-1.586	2.321
		X28	0.69	0.048	14.398	1.972	3.934	1.165	-0.974	0.166
		X29	0.793	0.035	22.718	2.662	4.089	1.054	-1.208	1.043
		X30	0.794	0.035	22.569	2.654	4.130	1.061	-1.233	0.991
		X31	0.815	0.037	22.062	2.984	4.478	0.847	-2.048	4.728
		X32	0.789	0.038	20.988	3.025	4.292	0.956	-1.593	2.500
		X33	0.787	0.042	18.876	2.987	4.324	0.884	-1.643	3.174
		X34	0.789	0.044	17.78	3.142	4.300	0.872	-1.465	2.588
		X35	0.653	0.049	13.443	1.905	4.035	1.076	-1.038	0.489
	Knowledge	X36	0.88	0.02	43.853	2.432	4.334	0.848	-1.593	3.279
		X37	0.783	0.044	17.878	1.777	3.881	1.133	-0.878	0.059
		X38	0.878	0.024	36.669	2.401	4.270	0.909	-1.542	2.643
		X39	0.85	0.026	32.176	2.136	3.934	1.012	-0.823	0.309
	Attitude	X40	0.772	0.04	19.484	2.158	4.073	1.026	-1.139	0.970
		X41	0.842	0.025	33.397	2.922	4.029	1.052	-1.131	0.891
		X42	0.786	0.041	19.402	2.272	4.251	0.962	-1.591	2.577
		X43	0.789	0.035	22.34	2.385	3.960	1.087	-1.078	0.736
		X44	0.781	0.042	18.512	2.173	3.942	1.040	-0.963	0.574
		X45	0.78	0.032	24.647	2.01	3.889	1.121	-1.008	0.404
		X46	0.702	0.039	18.169	2.27	3.353	1.300	-0.390	-0.919
		X47	0.681	0.048	14.172	2.216	3.417	1.208	-0.387	-0.746
	Skill	X48	0.829	0.024	33.97	1.912	3.551	1.117	-0.540	-0.348
		X49	0.85	0.023	37.164	2.16	3.728	1.111	-0.687	-0.243
		X50	0.823	0.031	26.587	1.929	3.867	1.077	-0.882	0.176
		X51	0.766	0.047	16.151	1.59	4.046	0.928	-0.836	0.504
	Family	X52	0.837	0.028	29.419	2.582	3.953	1.025	-1.021	0.786
		X53	0.844	0.028	29.997	2.691	4.168	0.959	-1.496	2.432
		X54	0.801	0.034	23.35	2.154	3.804	1.097	-0.885	0.295
		X55	0.841	0.03	28.124	2.554	4.032	1.002	-1.095	0.979
		X56	0.843	0.027	31.079	2.448	3.988	0.994	-1.115	1.224
		X57	0.82	0.033	24.716	2.326	3.991	1.047	-1.095	0.968
	Relatives and friends	X58	0.831	0.021	40.104	2.014	3.284	1.369	-0.351	-1.030
		X59	0.885	0.018	47.982	2.757	3.317	1.294	-0.346	-0.911
		X60	0.821	0.029	27.96	2.05	3.186	1.278	-0.201	-0.960
		X61	0.81	0.024	34.315	1.69	3.464	1.249	-0.433	-0.687
	Classmate	X62	0.746	0.035	21.12	1.992	3.316	1.288	-0.351	-0.888
		X63	0.695	0.04	17.286	1.957	3.194	1.314	-0.208	-1.058
		X64	0.701	0.037	19.076	1.645	2.630	1.298	0.358	-0.952
		X65	0.778	0.033	23.744	1.97	3.159	1.277	-0.174	-0.953
		X66	0.765	0.033	23.332	1.812	3.294	1.247	-0.302	-0.843
		X67	0.748	0.031	24.034	2.551	3.633	1.136	-0.676	-0.209
		X68	0.676	0.042	16.135	2.122	3.649	1.129	-0.570	-0.409
Second Order Constructs	Academic factors	Culture	0.909	0.014	64.465	-	3.883	0.757	-1.241	2.251
		Extracurricular activities	0.867	0.016	53.337	-	3.655	0.954	-0.823	0.552
		Facilities and equipment	0.723	0.041	17.684	-	3.821	0.914	-1.116	1.359
		Teaching-learning environment	0.934	0.009	102.273	-	4.184	0.785	-1.888	4.571
	Individual factors	Attitude	0.958	0.007	130.416	-	3.856	0.790	-1.168	2.155
		Knowledge	0.89	0.018	48.801	-	4.089	0.826	-1.428	3.013
		Skill	0.876	0.023	37.499	-	3.800	0.867	-0.814	0.722
	Social factors	Classmate	0.885	0.017	51.473	-	3.273	0.938	-0.313	-0.236
		Family	0.878	0.016	56.47	-	3.979	0.844	-1.244	2.144
		Relatives and friends	0.832	0.023	36.319	-	3.321	1.089	-0.370	-0.488
	Academic enthusiasm	Academic factors	0.917	0.018	50.876	-	3.884	0.734	-1.424	2.924
		Individual factors	0.916	0.012	75.106	-	3.911	0.731	-1.215	2.778
		Social factors	0.81	0.031	25.843	-	3.519	0.824	-0.626	0.679

Divergent validity is the extent to which a construct is correctly distinguishable from other constructs by an empirical measure. This validity was measured by the Fronell-Larcker criterion, according to which the square root of AVE for each latent construct must be greater than the highest correlation between that construct and other constructs. In other words, the square roots of AVE for latent constructs, which are positioned on the main diagonal of the matrix, should be higher than their correlations, which are positioned below this diagonal. The underlying logic of this requirement is that each construct must be able to explain the variance of its own indicators better than the variance of other constructs. As the results of Table 3 shows, all variables were found to have acceptable divergent validity.

**Table 3:** Construct Reliability and Validity

Construct	1	2	3	4	5	6	7	8	9	10
Attitude	0.768									
Classmate	0.568	0.731								
Culture	0.527	0.404	0.783							
Extracurricular activities	0.579	0.552	0.711	0.78						
Facilities and equipment	0.349	0.258	0.662	0.492	0.808					
Family	0.682	0.619	0.494	0.531	0.272	0.831				
Knowledge	0.779	0.54	0.684	0.631	0.462	0.651	0.849			
Relatives and friends	0.49	0.677	0.231	0.347	0.153	0.591	0.405	0.837		
Skill	0.77	0.617	0.514	0.547	0.286	0.681	0.687	0.46	0.818	
Teaching-learning environment	0.718	0.483	0.784	0.773	0.535	0.626	0.807	0.353	0.642	0.781

**Fig. 2:**
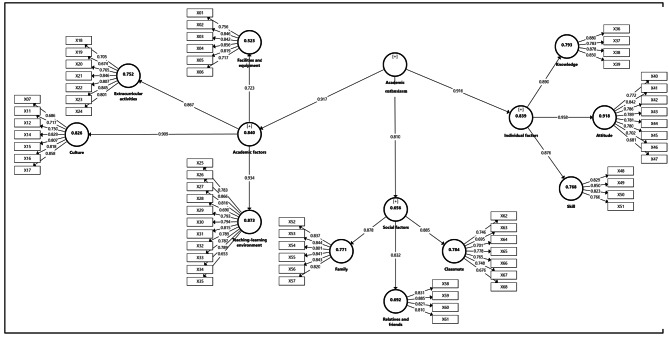
Paths Standardized coefficient (β)

Confirmatory factor analysis (CFA) was used to determine what factors influence academic enthusiasm from the students’ point of view. Figure 2 shows the two-level CFA model for standard coefficient estimation. The main variable in this model is “Academic Enthusiasm”, which has three dimensions: 1- University (consisting of four components: university facilities and equipment, university atmosphere, extracurricular activities, and teaching-learning environment), 2- Individual (knowledge, attitude, and skills) and 3- Social (family, relatives and acquaintances, friends, and classmates). Overall, this model consists of one main variable, 3 dimensions, 10 components, and 64 indicators.

To assess the validity of the measurement models, the following measures were calculated and compared with the requirements listed in Table 4. This assessment showed the acceptable validity of the measurement model.

As shown in Table 4, Cronbach’s alphas for all variables were above the acceptability threshold (0.6), indicating good reliability. The composite reliability coefficient (Dillon-Goldstein’s rho) for all variables was also above 0.7, suggesting good composite reliability. One measure of convergent validity is AVE, which is the average shared variance between the latent variable and its indicators and must be at least 0.50 for convergent validity to be acceptable. For this model, this measure was above 0.5, indicating acceptable convergent validity, for all variables. Another measure of convergent validity is Rho-A, which must be above 0.6 for convergent validity to be acceptable. This index was also found to be higher than the permissible limit for all research variables.

**Table 4:** Fitness of model

Variable	Cronbach’s Alpha	Rho_A	CR	AVE
Academic enthusiasm	0.974	0.977	0.975	0.583
Academic factors	0.962	0.964	0.965	0.571
Attitude	0.900	0.903	0.920	0.590
Classmate	0.854	0.856	0.889	0.534
Culture	0.894	0.901	0.917	0.613
Extracurricular activities	0.892	0.897	0.915	0.608
Facilities and equipment	0.892	0.896	0.918	0.652
Family	0.910	0.911	0.931	0.691
Individual factors	0.943	0.945	0.949	0.542
Knowledge	0.870	0.879	0.911	0.720
Relatives and friends	0.858	0.859	0.904	0.701
Skill	0.834	0.837	0.890	0.669
Social factors	0.930	0.932	0.938	0.573
Teaching-learning environment	0.935	0.938	0.945	0.611

Reliability(CR > 0.7 & Cronbach’s Alpha > 0.6); Convergent validity(CR > AVE, AVE > 0.5, Rho_A > 0.6)

Since the designed model was a measurement model, one-sample t-test was used to investigate the impact of dimensions and indicators on academic enthusiasm from the perspective of the studied community. Considering that all questions of the questionnaire were designed directly, if the average obtained in the range of one to five was less than 3, it means no effect, and if it was more than 3, the effect is significant. In fact, the value of the norm score is usually considered the middle of the spectrum 3. The results of Table 5 show that for all dimensions, the average value of the sample was greater than 3 and the value of the significance level was less than 0.05 (t > 1.96), which means that all factors are effective on academic enthusiasm.

**Table 5:** One-sample t-test results to investigate the impact of dimensions and components on academic enthusiasm

Construct	Mean	Std. Deviation	Test Value = 3			
			t	Sig	Lower	Upper
Facilities and equipment	3.821	0.914	16.937	0.000	0.726	0.917
Culture	3.883	0.757	21.987	0.000	0.804	0.962
Extracurricular activities	3.655	0.954	12.914	0.000	0.555	0.755
Teaching-learning environment	4.184	0.785	28.348	0.000	1.102	1.267
Knowledge	4.089	0.826	24.676	0.000	1.002	1.176
Attitude	3.856	0.790	20.311	0.000	0.773	0.938
Skill	3.800	0.867	17.304	0.000	0.709	0.891
Family	3.979	0.844	21.751	0.000	0.890	1.067
Relatives and friends	3.321	1.089	5.512	0.000	0.206	0.435
Classmate	3.273	0.938	5.482	0.000	0.175	0.371
Academic factors	3.884	0.734	22.699	0.000	0.807	0.961
Individual factors	3.911	0.731	23.424	0.000	0.834	0.987
Social factors	3.519	0.824	11.868	0.000	0.433	0.605

According to the results obtained from the analysis of variance (MANOVA) presented in Table 6, there is a significant difference between the opinions of men and women regarding the impact of Skill, Family and Individual factors on academic enthusiasm at the 95% confidence level (P < 0.05).

According to the effect size index, the difference between the two groups was 1.2%, 1.9% and 1.2%, respectively, and based on the observed averages, from the point of view of women, the intensity of the effect of these three variables on academic enthusiasm was greater than that of men.

**Table 6:** Comparing the opinions of men and women using multivariate analysis of variance (MANOVA)

Construct	Gender	Mean	Std. Deviation	N	F	Sig	Effect Size	Result
Facilities and equipment	Female	3.898	0.901	208	2.945	0.087	0.008	not significant
	Male	3.726	0.930	138				
	Total	3.829	0.915	346				
Culture	Female	3.928	0.726	208	0.499	0.480	0.001	not significant
	Male	3.871	0.748	138				
	Total	3.905	0.734	346				
Extracurricular activities	Female	3.667	0.934	208	0.092	0.762	0.000	not significant
	Male	3.698	0.927	138				
	Total	3.680	0.930	346				
Teaching-learning environment	Female	4.192	0.743	208	0.450	0.503	0.001	not significant
	Male	4.246	0.733	138				
	Total	4.214	0.738	346				
Knowledge	Female	4.154	0.803	208	1.942	0.164	0.006	not significant
	Male	4.030	0.816	138				
	Total	4.105	0.809	346				
Attitude	Female	3.939	0.716	208	3.586	0.059	0.010	not significant
	Male	3.779	0.853	138				
	Total	3.875	0.776	346				
Skill	Female	3.888	0.792	208	4.169	0.042	0.012	significant difference
	Male	3.698	0.929	138				
	Total	3.812	0.853	346				
Family	Female	4.083	0.778	208	6.696	0.010	0.019	significant difference
	Male	3.847	0.905	138				
	Total	3.989	0.838	346				
Relatives and friends	Female	3.310	1.075	208	0.118	0.732	0.000	not significant
	Male	3.351	1.130	138				
	Total	3.327	1.096	346				
Classmate	Female	3.283	0.947	208	0.003	0.955	0.000	not significant
	Male	3.277	0.923	138				
	Total	3.281	0.936	346				
Academic factors	Female	3.921	0.713	208	0.211	0.646	0.001	not significant
	Male	3.885	0.709	138				
	Total	3.907	0.710	346				
Individual factors	Female	3.994	0.677	208	4.151	0.042	0.012	significant difference
	Male	3.835	0.751	138				
	Total	3.931	0.711	346				
Social factors	Female	3.559	0.787	208	0.558	0.456	0.002	not significant
	Male	3.492	0.856	138				
	Total	3.532	0.814	346				

## Discussion

This study aimed to identify and examine the factors that influence the academic enthusiasm of the students of a university of medical sciences with the purpose of improving the university’s pedagogy paradigm.

The results of the study showed that 3 dimensions (academic, individual and social), 10 components (teaching-learning environment, culture, extracurricular activities, facilities and equipment, attitude, knowledge, skill, classmate, family, relatives and friends) and 64 indicators were effective in measuring the main variable of students’ academic enthusiasm. This is consistent with the results of Nekavand et al. (2018), who found that academic enthusiasm is influenced by individual factors and university factors [28], and also with the results of Mirzaei et al. (2019), who stated that academic enthusiasm increases with the increase of social support (from family, friends, and others) [29]. In explaining this finding, it can be said that academic enthusiasm is a multi-dimensional construct, and multi-dimensional cognitive-behavioral interventions are needed to improve the paradigm of managers and educational policy makers.

The weight created by the latent variables Academic factors (Rsquare = 0.84) and Individual factors (Rsquare = 0.839) in measuring the main variable of academic enthusiasm is more than the weight created by social factors (Rsquare = 0.656). Academic factors such as the modus operandi of educational institutions has a profound impact on the quality of education they provide [30]. To achieve better results in the field of education, academics need to engage more actively with their education role [2]. Its necessary university staff and professors need to treat and engage with students without any form of discrimination or bias. By accepting and respecting students, a university can inspire students to not only strive for greater academic accomplishment but also improve their social relations and human ethics. Academic factors included four components: teaching and learning environment, culture, extracurricular activities, facilities and equipment. It seems necessary for the administrators and educational policymakers of this university to provide a happy and open educational environment. Academic factors included four components: teaching and learning environment, culture, extracurricular activities, facilities and equipment. The weight of the teaching and learning environment component (Rsquare = 0.873) was higher than the others. An environment governed by reasonable disciplinary rules and respectful of diversity to attract students to the greatest extent possible and provide the basis for their satisfactory participation in all areas of education. An environment with a friendly and free-thinking communication atmosphere so that students can express their opinions. Also, giving challenging assignments to students has a positive effect on their participation in university activities. A university’s atmosphere is greatly influenced by the support and commitment of its senior management [31]. One of the major duties of education managers is to motivate students to achieve the highest possible performance level [32]. Performance is directly influenced by leadership variables, organizational culture, work motivation, and job satisfaction. In the context of TQM, the concept of leadership has been described as the ability to motivate others by providing inspiration, motivation, or enthusiasm [33]. Thus, it can be concluded that effective leadership plays a role not only in providing excellent services in the healthcare system [34] but also in providing quality educational services in the higher education system through the fulfillment of the conditions and requirements for increasing the students’ academic enthusiasm.

In terms of importance, individual factors was the second most effective factor on academic enthusiasm, it included three components of attitude, knowledge and skill. The weight of the attitude component (Rsquare = 0.918) was higher than the others.

Regarding the finding that academic enthusiasm is influenced by individual factors, it can be argued that students develop a positive or negative attitude towards themselves and their environment over the course of their education; an attitude that strongly depends on whether they have had successful or unsuccessful experiences during this time. In fact, they interact with a wide variety of factors that affect their cognitive, emotional, and social development during the time that many consider to be the best years of their lives. The purpose of all education courses is to help students acquire knowledge, skills, and attitudes that they will need in their profession. It can be argued that to gain mastery in an area, in addition to acquiring the mental and motor skills required for the task, it is also crucial to acquire the attitude needed for success in that field of activity. Therefore, people’s attitude towards education is of great importance for their performance and success. A student with a positive attitude towards university and education will be more likely to study with great enthusiasm. Students with superior individual characteristics like knowledge, attitude, and skills (compared to other students) tend to be more successful in maintaining their motivation, perseverance, and effort levels in the face of difficulties and obstacles and as a result, achieve higher academic performance. Therefore, it is necessary for the administrators and educational policy makers of this university to interview the students several times and get to know their attitudes by holding meetings. Also, activate the students’ advisory committee more. Creating an atmosphere of higher academic enthusiasm requires involving students in their own learning [35]. Student counselors can also be effective in identifying factors that facilitate learning for all students [36].

The third effective factor known in this research was social factors, including family, classmate, relatives and friends. Therefore, it is necessary to create a link between the university and the family through the ideas and opinions of the families and send the feedback to the single cultural council to make decisions. Attracting the participation of supportive families and volunteers can be effective in solving the problems and issues of vulnerable student. Family as the social unit in which a person experiences group life, can greatly shape a person’s educational development and response to social rules. A family’s disciplinary, moral, economic, and emotional conditions may prepare a person to live better and better overcome life challenges, thereby helping them achieve greater educational accomplishment, or on the contrary, prevent them from realizing their education and development potentials. Since it is very difficult to be a good student in a turbulent and unsafe home, parents can facilitate their children’s academic development by maintaining a peaceful environment at home. Among these components, classmate (Rsquare = 0.784) had more weight. Interaction with peers and classmates who are interested in education can trigger a sense of competition that can help increase a student’s academic enthusiasm. On the contrary, students who have a negative attitude towards education and university can induce a similar attitude and feeling in their peers.

The findings of this study are consistent with the results of Grohman and Snyder [37] and Izadpanah and Rezaei [38], which showed that teacher-learner interaction, is an indirect predictor of academic enthusiasm. Also, Students learn all manners of social rules, norms, and principles initially from their family and then from their relatives, acquaintances, friends, and classmates. Social support also helps people endure psychological pressures and even resolve them with the help of others. Social support is a well-known coping force for dealing with conflict and tension, which greatly helps people overcome challenging tasks. A person who does not feel alone in the face of challenges and can count on the help and support of others in such situations will be less likely to exhibit weakness and despair and will be more capable of mustering personal strengths as well as seeking help from others when encountering problems.

In the present study, another category of factors that were found to be affecting academic enthusiasm were social factors. This is consistent with the results of Eslami et al. (2016), who found that academic enthusiasm is influenced by social factors (supported academic performance, supportive relationships with peers, and family support) and individual factors (self-efficacy, optimism, and self-esteem) [39]. This finding is consistent with the results of Ansong [40]. According to the results obtained from the analysis of variance (MANOVA), there is a significant difference between men’s and women’s opinions on the impact of Skill, Family and Individual factors on academic enthusiasm.

From the point of view of women, the intensity of the effect size of three variables on academic enthusiasm was more than that of men. Perhaps in explaining this finding, it can be said that women spend more time with their families than men [41, 42]. Unlike women, men do not pay much attention to details and do not look at the whole. Considering that academic enthusiasm is a complex multi-dimensional phenomenon, any solution devised for improving students’ academic enthusiasm should also be multi-dimensional.

Based on the findings of this study, education managers who shape the university’s pedagogy are recommended to pay more attention to the principles of organizational equity including fairness and equality in the allocation of facilities, achievements, responsibilities, resources, and educational programs and tasks, and also fairness and equality, respect, and honesty when communicating and dealing with students. Considering the importance of teaching methods and the fast evolution of educational knowledge in the field of medical science, the university should create better mechanisms for the improvement of faculty members in this respect through experience (e.g. providing study opportunities in the field of education, adopting new teaching methods, and using novel educational technologies). University should be a place where students are applauded for their achievements and are encouraged to realize their potentials. Student counselors can play a key role in the creation of such an atmosphere by implementing reward programs that recognize and celebrate students’ successes (e.g. for being valedictorian or salutatorian). Some students appear to be not satisfied with the structure and requirements of university programs and find it difficult to conform to the university environment and the expectations of others. Counselors should also pay more attention to students who are struggling academically or are constantly trying to just get through the day. These counselors can enhance the students’ academic enthusiasm by building a healthy space for effective learning and counseling interventions with the help of parents, professors, and other university employees. 

Some limitations of this research should be considered. The study was performed in one university; therefore, findings may not be generalizable to other context. Further research done at other universities is recommended. Collaborative research across universities could be very helpful. Self-filling of the questionnaires creates the possibility of bias in the selection. In the study, the generalization of the results to other universities of medical sciences should be done with caution.

## Conclusion

Identification of the factors that influence students’ academic enthusiasm allows education managers, as the most important actors in the shaping of university pedagogy, to devise better plans for improving the quality of education provided at the university. The developed model can help medical universities take a step towards improving the academic productivity and performance of their students and gain a competitive advantage in this respect.

## Data Availability

The datasets are available from the corresponding author on reasonable request.

## Abbreviations

AVE: Average variance Extracted
CR: Construct Reliability
